# A Systematic Review and Meta‐Analysis to Evaluate the Effects of Chitosan on Obesity Indicators

**DOI:** 10.1002/fsn3.4596

**Published:** 2024-11-19

**Authors:** Mona Kholdebarin, Naseh Pahlavani, Mahlagha Nikbaf‐Shandiz, Halle Mosallaei, Niloufar Rasaei, Zeinab Khalse, Yasaman Aali, Omid Asbaghi, Ali Zamanian, Farideh Shiraseb

**Affiliations:** ^1^ Science and Research Branch Tehran Islamic Azad University Tehran Iran; ^2^ Health Sciences Research Center Torbat Heydariyeh University of Medical Sciences Torbat‐e Heydarieh Iran; ^3^ Student Research Committee Tabriz University of Medical Sciences Tabriz Iran; ^4^ Rehabilitation Research Center Iran University of Medical Sciences Tehran Iran; ^5^ Micronutrient Research Center, Research Institute for Endocrine Sciences Shahid Beheshti University of Medical Sciences Tehran Iran; ^6^ Department of Community Nutrition, School of Nutritional Sciences and Dietetics Tehran University of Medical Sciences (TUMS) Tehran Iran; ^7^ Department of Pharmacy, Faculty of Pharmacy University of Tehran Tehran Iran; ^8^ Department of Nutrition Faculty of Medicine, Mashhad University of Medical Sciences Mashhad Iran; ^9^ Cancer Research Center Shahid Beheshti University of Medical Sciences Tehran Iran; ^10^ Student Research Committee Shahid Beheshti University of Medical Sciences Tehran Iran; ^11^ Shahid Beheshti University of Medical Sciences Tehran Iran

**Keywords:** body composition, chitosan, meta‐analysis, obesity, systematic review

## Abstract

Chitosan, a commonly used dietary supplement, is believed to have the potential to decrease body weight by binding to dietary fats and decreasing their absorption. However, due to conflicting results from various studies, this review aimed to investigate the effects of chitosan supplementation on obesity indicators in adults. To find appropriate randomized clinical trials (RCTs), a thorough search was conducted across electronic databases like PubMed/Medline, Scopus, and ISI Web of Science. The random‐effects method was employed to combine the data, and the outcomes were presented as the weighted mean difference (WMD) with 95% confidence intervals (CIs). In total, 19 RCTs with 21 effect sizes were included in the meta‐analysis. The combined analysis showed that chitosan supplementation significantly reduced body weight (WMD = −0.79 kg; 95% CI, −1.30 to −0.29; *p* = 0.002) and body‐fat percentage (BFP) (WMD = −0.41%; 95% CI, −0.50 to −0.32; *p* < 0.001). Additionally, there was a notable increase in fat‐free mass (FFM) (WMD = 0.20 kg; 95% CI, 0.06–0.34; *p* = 0.005). However, no significant impact of chitosan on body mass index (BMI) (WMD = −0.35 kg/m^2^, 95% CI: −0.71, 0.00; *p* = 0.054) and waist circumference (WC) (WMD = −0.71 cm, 95% CI: −1.49, 0.05; *p* = 0.069) was observed. Overall, chitosan supplementation shows promise in improving obesity indicators by reducing BFP and increasing FFM. However, further well‐designed studies with larger sample sizes are needed to confirm these findings.

## Graphical abstract

Chitosan supplementation may improve obesity indicators by decreasing BFP and increasing FFM. Well‐methodological studies with greater sample sizes are still needed to verify obtained results.

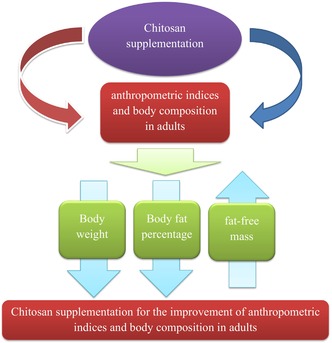

## 1 Introduction

Over the past decade, obesity has surged into a global epidemic, posing an increasingly significant public health challenge (Lingvay et al. 2024). This pervasive issue has prompted researchers to utilize various indices for assessing obesity prevalence, including body mass index (BMI), waist circumference (WC), body‐fat percentage (BFP), and skin fold thickness (Nimptsch, Konigorski, and Pischon 2019). Obesity is intricately linked with a myriad of health complications, ranging from type 2 diabetes mellitus to cardiovascular complications such as arteriosclerosis, dyslipidemia, and hypertension (Malnick and Knobler 2006).

The escalating prevalence of obesity has led to a deeper understanding of its multifaceted impacts on public health. As societies grapple with the complex interplay of genetic, environmental, and behavioral factors contributing to obesity, interventions targeting its prevention and management have become increasingly imperative. Initiatives focusing on lifestyle modifications, dietary interventions, and promoting physical activity have gained traction as pivotal strategies in combating the obesity epidemic.

The consumption of functional and bioactive foods has emerged as a promising avenue for enhancing individual health (Asbaghi et al. 2022; Zamani et al. 2023, 2022). Among these, chitosan oligosaccharides, derived from chitin and extracted from crustaceans such as crabs (Shepherd, Reader, and Falshaw 1997), have garnered significant attention in recent research for their diverse biological roles. Studies have highlighted their potential to enhance body weight, reduce body fat, modulate blood glucose and insulin resistance, and exhibit anti‐tumor and antioxidant properties (Eleanor Barrager, Schauss, and Nichols 2001; Mengíbar et al. 2013).

Numerous clinical investigations have underscored the association between chitosan consumption and improvements in anthropometric and body composition measures (Hernández‐González et al. 2010; Trivedi et al. 2016). However, it is essential to note that conflicting findings exist within the literature. While some studies have reported significant beneficial effects of chitosan on these parameters, others have failed to observe such effects. (Bokura and Kobayashi 2003; Pittler et al. 1999).

The impact of chitosan on body weight has garnered considerable attention, with studies suggesting its ability to elevate leptin levels as an appetite suppressant and reduce fat absorption in the intestine due to its poor emulsification properties (Gades and Stern 2003). Meta‐analyses conducted by Mhurchu et al. (2005) and Huang et al. (2020) demonstrated statistically significant decreases in body weight among overweight and obese individuals who supplemented with chitosan. Additionally, a recent meta‐analysis by Perna et al. (2020) highlighted the potential of polyglucosamine, a low‐molecular‐weight chitosan variant, to result in decreases in anthropometric measures such as body weight, BMI, and WC in overweight or obese individuals (Perna et al. 2020).

Since a number of qualified articles have been missed in these meta‐analyses and also a number of new randomized clinical trials (RCTs) have been published after these meta‐analyses, there is a need for a comprehensive and up‐to‐date systematic review and meta‐analysis with broader statistical analysis.

## 2 Methods

The current study followed the Preferred Reporting Items for Systematic Reviews and Meta‐Analyses (PRISMA) guidelines (Moher et al. 2009). The present study was registered at PROSPERO (CRD42022358601).

### 2.1 Search Strategy

To assess the effects of chitosan supplementation on anthropometric indicators and body composition, a systematic search was conducted in electronic databases including PubMed/Medline, Scopus, and ISI Web of Science up to August 2024. The search utilized specific keywords to identify relevant studies: (“Chitosan”[Title/Abstract] OR “Chitin”[Title/Abstract] OR “Poliglusam”[Title/Abstract]) AND (Intervention[Title/Abstract] OR “controlled trial”[Title/Abstract] OR randomized[Title/Abstract] OR randomized[Title/Abstract] OR random[Title/Abstract] OR randomly[Title/Abstract] OR placebo[Title/Abstract] OR “clinical trial”[Title/Abstract] OR blinded[Title/Abstract] OR trials[Title/Abstract] OR “Cross‐Over”[Title/Abstract] OR parallel[Title/Abstract]). The participant, intervention, comparison, and outcome (PICO) search framework was utilized to identify relevant studies linked to chitosan supplementation, anthropometric indicators, and body composition. To ensure comprehensive coverage, we conducted a snowball search; hence, a manual search was also conducted in Google Scholar, and the reference lists of included studies were reviewed to avoid missing any eligible studies.

### 2.2 Study Selection

Screening of articles based on titles and abstracts was conducted independently by two authors (FS and OA). Studies were selected according to the following PICOS criteria (Participant: overweight and obese adults, Intervention: chitosan, Comparison: control group, Outcome: body weight, BMI, WC, BFP, and fat‐free mass (FFM), Study design: RCT). Additionally, we included studies with intervention periods of no less than 4 weeks (considering randomized controlled trials with at least eligible arms as distinct findings) that provided means and standard deviations (SDs) for the evaluation of body weight, BMI, WC, BFP, FFM, or any other quantifiable factor presented as SD. The search encompassed human studies without language restrictions. Exclusion criteria consisted of (1) non‐randomized studies or RCTs lacking a control group, (2) animal and review studies, (3) research conducted on children or adolescents, and (4) dissertations, case reports, conference papers, editorial papers, and books.

### 2.3 Data Extraction

Two commentators, FS and OA, independently judged the RCTs for suitability. Papers not meeting the criteria were excluded. Extracted data included details such as the first author's name, publication year, country, gender, RCT design, health conditions, intervention type and dosage, duration of chitosan supplementation, participant compliance, and other characteristics such as mean age, and BMI. Additionally, mean and SD of outcomes before and after the intervention were documented. If specific information was lacking, the average change in values was computed. Moreover, if chitosan supplement dosage was reported in grams per day, it was changed to milligrams per day.

### 2.4 Quality Assessment

The Cochrane Collaboration tool was utilized to evaluate the quality of included papers (Higgins et al. 2011). Studies were assessed for any source of bias, such as random sequence generation, allocation concealment, blinding of participants and personnel, blinding of outcome assessment, incomplete outcome data, selective reporting, and other biases. Based on quality, studies were categorized into (1) uncertain risk of bias, (2) low risk of bias, and (3) high risk of bias (refer to Table 1). Quality assessment was performed by FS and OA.

**TABLE 1:** Characteristic of included studies in meta‐analysis.

Studies	Country	Study design	Participant	Sample size and sex	Sample size		Trial duration (week)	Mean age		Mean BMI		Intervention		Adverse events
					IG	CG		IG	CG	IG	CG	chitosan (mg/d)	Control group	
Pittler et al. 1999	United Kingdom	Parallel, R, PC, DB	Overweight volunteers	M/F: 30	15	15	4	46.7 ± 8.8	41.3 ± 11.3	26.3 ± 1.6	26.9 ± 1.8	1000	Placebo	No side effect
Schiller et al. 2001	USA	Parallel, R, PC, DB	Overweight and mildly obese individuals	F: 59	29	30	8	41 ± 8.2	40 ± 8.3	32.2 ± 4.8	31.8 ± 4.2	3000	Placebo	NR
Ho et al. 2001 (A)	Singapore	Parallel, R, PC, DB	Obese Asian subjects	F: 31	16	15	12	42.8 ± 6	44.3 ± 8.1	25.6 ± 2.6	24.6 ± 3.3	250	Placebo	Gastrointestinal included epigastric discomfort, constipation, diarrhea, nausea, and dryness of throat
Ho et al. 2001 (B)	Singapore	Parallel, R, PC, DB	Obese Asian subjects	M: 37	20	17	12	42.4 ± 7.3	42.5 ± 7.5	25.7 ± 3.7	27 ± 3.4	250	Placebo	Gastrointestinal included epigastric discomfort, constipation, diarrhea, nausea, and dryness of throat
Zahorska‐Markiewicz et al. 2002	Poland	Parallel, R, PC, DB	Obese but otherwise healthy females	F: 32	32	32	24	22–59	22–59	> 30	> 30	4500	Placebo	NR
Woodgate et al. 2003	Canada	Parallel, R, PC, DB	Obese adults	M/F: 24	12	12	6	35.4 ± 8.6	38.5 ± 7.9	36.8 ± 7.7	34.6 ± 4.4	1395	Placebo	Constipation, flatulence, increased defecation frequency, swelling, different kinds of pain, rash, heart palpitation, and insomnia
Bokura and Kobayashi et al. 2003	Japan	Parallel, R, PC, DB	Healthy women	F: 90	45	45	8	56.7 ± 8	56.4 ± 9	NR	NR	1200	Placebo	Dry mouth, insomnia, anorexia, and constipation
Metso et al. 2003	Finland	Crossover, R, PC, DB	Subjects with increased plasma total cholesterol	M/F: 130	130	130	12	32–64	32–64	26.9 ± 3.7	25.8 ± 3.3	2400	Placebo	Thirsty, oral aphta, abdominal fullness, and headache
Mhurchu et al. 2004	New Zealand	Parallel, R, PC, DB	Overweight and obese adults	M/F: 250	125	125	24	47 ± 11.7	48 ± 11.5	34.8 ± 5.1	36 ± 5.1	3000	Placebo	Non‐infectious gastrointestinal side effects defined as abdominal pain, bloating, constipation, indigestion, or non‐infectious
Lehtimäki et al. 2005	Finland	Crossover, R, PC, DB	Overweight volunteers	M/F: 96	35	61	12	44 ± 8.5	43.7 ± 9.7	26.1 ± 3.5	26.6 ± 3.5	2400	Placebo	No side effect
Kaats et al. 2006	USA	Parallel, R, PC, DB	Overweight volunteers	M/F: 88	46	42	8	43.9 ± 11.6	48.7 ± 11.6	NR	NR	3000	Placebo	No side effect
Liao et al. 2007 (A)	Taiwan	Parallel, R, PC, SB	Hyperlipidemic patients	M/F: 40	20	20	8	61.1 ± 11.25	63.9 ± 11.7	27.1 ± 4.05	27.6 ± 4.05	300	Placebo	NR
Liao et al. 2007 (B)	Taiwan	Parallel, R, PC, SB	Hyperlipidemic patients	M/F: 40	20	20	8	61.6 ± 12.15	63.9 ± 11.7	26.9 ± 3.6	27.6 ± 4.05	300	Placebo	NR
Hernández‐González et al. 2010	Mexico	Parallel, R, PC, DB	Obese subjects	M/F: 12	6	6	12	41.6 ± 6.3	42.6 ± 5.6	34.3 ± 2.7	32.7 ± 1.7	750	Placebo	No side effect
Willers et al. 2012	Germany	Parallel, R, PC, DB	Obese subjects	M/F: 106	52	54	12	45.4 ± 7	48.3 ± 7.1	31.7 ± 2	31.7 ± 1	800	Placebo	Common cold, hypertriglyceridemia, body ache, constipation (two subjects), and hypertension
jung et al. 2014	South Korea	Parallel, R, PC, DB	Overweight women	F: 53	53	53	8	23.3	23.3	24.2	24.2	3000	Placebo	NR
Trivedi et al. 2015	India	Parallel, R, PC, SB	Obese subjects	M/F: 96	64	32	12	35.53 ± 11.23	36.28 ± 10.49	30.93 ± 2.69	30.91 ± 2.72	500	Placebo	Common cold, hypertriglyceridemia, body ache, constipation, and hypertension
Pokhis et al. 2015	Germany	Parallel, R, PC, DB	Obese subjects	M/F: 87	45	42	24	48.6 ± 8.67	50 ± 8.91	35.1 ± 3.73	35.4 ± 3.62	1700	Placebo	No side effect
Santas et al. 2017	Spain	Parallel, R, PC, DB	Overweight and obese adults	M/F: 56	25	31	12	51.3 ± 10	45.9 ± 12	29.1 ± 3	29.2 ± 2.4	3000	Placebo	Increase of flatulence
Cornelli et al. 2017	Italy	Parallel, R, PC, DB	Overweight and obesity with polyglucosamine	M/F: 97	49	48	52	47 ± 7.75	46.4 ± 4.42	33.9 ± 1.03	34.1 ± 1.03	1600	Placebo	NR
Lütjohann et al. 2018	Germany	Parallel, R, PC, DB	Obese subjects	M/F: 116	61	55	12	18–65	18–65	31.8 ± 2.3	31.6 ± 2.3	3200	Placebo	NR

Abbreviations: CG, control group; CO, controlled; DB, double‐blinded; F, female; IG, intervention group; M, male; NR, not reported; PC, placebo‐controlled; RA, randomized; SB, single‐blinded.

### 2.5 Statistical Analysis

In this study, the overall effect size was determined using mean and SD changes. SD changes were calculated based on the following formula (Borenstein et al. 2011):

SDchange=[SDbaseline^2+SDfinal^2−2R×SDbaseline×SDfinal

In the formula, we considered the correlation coefficient (*R*) as a constant number equal to 0.8. If SE was used in the studies, the formula SD = SE × √*n* (*n* = the number of individuals in each group) was utilized to convert standard errors (SEs) to SD (Hozo, Djulbegovic, and Hozo 2005). The outcomes were given as the weighted mean difference (WMD) and 95% confidence interval (CI) for all effect sizes. Heterogeneity among studies was evaluated using Cochrane's *Q* test (*p* < 0.1 considered significant) and the *I* ^2^ test, with the fixed‐effects model being utilized when *I* ^2^ was less than 40% (DerSimonian and Kacker 2007; Higgins et al. 2003). Subgroup analysis was employed to distinguish the underlying cause of heterogeneity. The selection of subgroups was guided by the minimum number of studies required based on the specified criteria outlined by Fu et al. Specifically, a minimum of 6–10 studies were needed for continuous variables, and at least four studies for categorical subgroup variables (Fu et al. 2011; Research and Quality 2011). Subgroup analyses were performed based on baseline BMI (overweight: 25–29.9, obese: ≥ 30 kg/m^2^), dose (< 1500, ≥ 1500 mg/day), and duration (< 12, ≥ 12 weeks) of the intervention. A funnel plot test was used for publication bias (Begg and Mazumdar 1994; Egger et al. 1997). To assess the impact of each study on the pooled‐effect size, the leave‐one‐out method (i.e., excluding one trial at a time and recalculating the effect size) and sensitivity analysis were directed. Meta‐regression analysis was performed to evaluate the influence of dosage and the duration of chitosan supplementation (mg/day) on body weight, BMI, WC, BFP, and FFM. Additionally, non‐linear regression was utilized to examine the non‐linear relationship between chitosan and anthropometric indicators and body composition.

### 2.6 Certainty Assessment

The researchers (OA and MK) utilized the GRADE (Appraisal, Development, and Evaluation of Recommendations) approach to assess and consolidate the overall certainty of the studies included in the review (Guyatt et al. 2008).

## 3 Results

### 3.1 The Flow Diagram of the Study Selection

**FIGURE 1:**
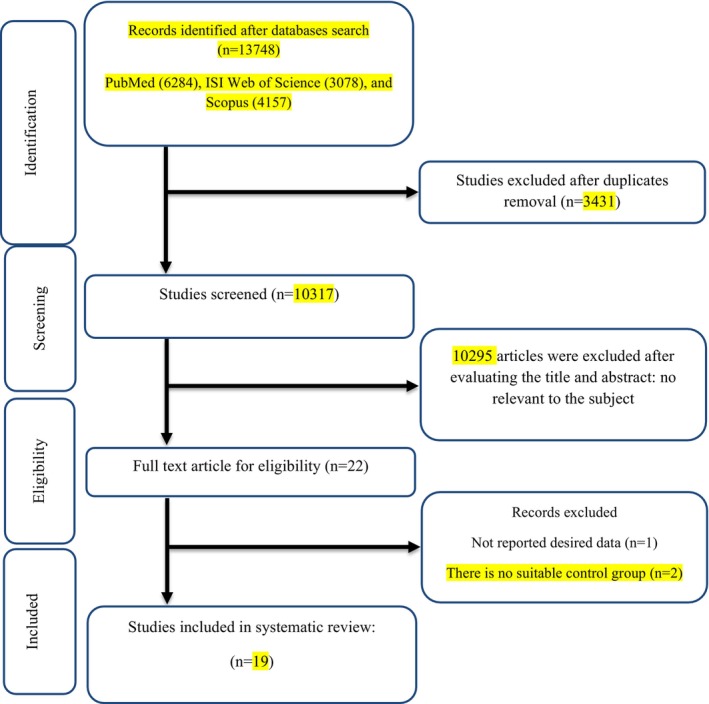
Flow chart of study selection for inclusion trials in the systematic review.

The study flow chart, illustrated in Figure 1, began with the identification of 13,748 studies through electronic database searches. Among these, 3431 duplicates and 10,295 irrelevant studies were excluded based on titles and abstracts. Following the review of 22 full‐text relevant articles, three studies were excluded for not reporting desired data (Macchi 1996; Stoll, Bitterlich, and Cornelli 2017). Finally, 19 studies were included in the quantitative synthesis (Bokura and Kobayashi 2003; Cornelli et al. 2017; Hernández‐González et al. 2010; Ho et al. 2001; Jung et al. 2014; Kaats, Michalek, and Preuss 2006; Lehtimäki et al. 2005; Liao et al. 2007; Lütjohann et al. 2018; Metso et al. 2003; Mhurchu et al. 2004; Pittler et al. 1999; Pokhis et al. 2015; Santas, Lázaro, and Cuñé 2017; Schiller et al. 2001; Trivedi et al. 2015; Willers, Plötz, and Hahn 2012; Woodgate and Conquer 2003; Zahorska‐Markiewicz et al. 2002).

### 3.2 Study Characteristics

The included studies were conducted in Japan (Bokura and Kobayashi 2003), United Kingdom (Pittler et al. 1999), Singapore (Ho et al. 2001), Finland (Lehtimäki et al. 2005; Metso et al. 2003), Canada (Woodgate and Conquer 2003), New Zealand (Mhurchu et al. 2004), USA (Kaats, Michalek, and Preuss 2006; Schiller et al. 2001), Taiwan (Liao et al. 2007), Mexico (Hernández‐González et al. 2010), Germany (Lütjohann et al. 2018; Pokhis et al. 2015; Willers, Plötz, and Hahn 2012), India (Trivedi et al. 2015), Spain (Santas, Lázaro, and Cuñé 2017), Poland (Zahorska‐Markiewicz et al. 2002), and South Korea (Jung et al. 2014) from 1996 to 2018. The study characteristics are detailed in Table 1. There were 19 parallel (Bokura and Kobayashi 2003; Cornelli et al. 2017; Hernández‐González et al. 2010; Ho et al. 2001; Jung et al. 2014; Kaats, Michalek, and Preuss 2006; Liao et al. 2007; Lütjohann et al. 2018; Mhurchu et al. 2004; Pittler et al. 1999; Pokhis et al. 2015; Santas, Lázaro, and Cuñé 2017; Schiller et al. 2001; Trivedi et al. 2015; Willers, Plötz, and Hahn 2012; Woodgate and Conquer 2003; Zahorska‐Markiewicz et al. 2002) and two crossover studies (Lehtimäki et al. 2005; Metso et al. 2003). The study participants' mean age ranged from 18 to 65 years, with baseline BMI varying from 24.2 to 36.8 kg/m^2^. Chitosan supplementation duration in the included papers ranged from 4 to 52 weeks, and daily dosages ranged from 135 to 4500 mg. All studies, except for five, included participants of both sexes (Bokura and Kobayashi 2003; Ho et al. 2001; Jung et al. 2014; Schiller et al. 2001; Zahorska‐Markiewicz et al. 2002). Studies included participants who were overweight and obese (Cornelli et al. 2017; Hernández‐González et al. 2010; Ho et al. 2001; Jung et al. 2014; Kaats, Michalek, and Preuss 2006; Lehtimäki et al. 2005; Lütjohann et al. 2018; Mhurchu et al. 2004; Pittler et al. 1999; Pokhis et al. 2015; Santas, Lázaro, and Cuñé 2017; Schiller et al. 2001; Trivedi et al. 2015; Willers, Plötz, and Hahn 2012; Woodgate and Conquer 2003; Zahorska‐Markiewicz et al. 2002), healthy women (Bokura and Kobayashi 2003), and patients with hypercholesterolemia (Liao et al. 2007; Metso et al. 2003). In the investigation by Ho et al. (2001), two types of sex with different sample sizes [male (*n* = 37) and female (*n* = 31)] were enrolled. Therefore, two arms for this study were considered. Also, in a clinical trial by Liao et al. (2007), two types of intervention (water‐soluble chitosan and water‐insoluble chitosan) were used, so we considered two arms for this study. Nineteen effect sizes were considered for the effect of chitosan supplementation on weight, 19 effect sizes for BMI, 10 effect sizes for WC, eight effect sizes for BFP, and five effect sizes for FFM.

### 3.3 Adverse Events

Data on adverse events was stated in the studies by Ho et al. (2001) (gastrointestinal‐related side effects including epigastric discomfort, constipation, diarrhea, nausea, and dryness of throat), Trivedi et al. (2015) (common cold, hypertriglyceridemia, body ache, constipation, and hypertension), Willers, Plötz, and Hahn (2012) (common cold), Mhurchu et al. (2004) (non‐infectious gastrointestinal side effects defined as abdominal pain, bloating, constipation, indigestion, or non‐infectious diarrhea), Metso et al. (2003) (constipation, flatulence, increased defecation frequency, swelling, different kinds of pain, rash, heart palpitation, and insomnia), Santas, Lázaro, and Cuñé (2017) (an increase of flatulence), Bokura et al. (Santas, Lázaro, and Cuñé 2017) (thirsty, oral aphtha, abdominal fullness, and headache), and Woodgate and Conquer (2003) (dry mouth, insomnia, anorexia, and constipation). Table 2 displays the adverse events.

**TABLE 2:** Risk of bias assessment.

Studies	Random sequence generation	Allocation concealment	Selective reporting	Other sources of bias	Blinding (participants and personnel)	Blinding (outcome assessment)	Incomplete outcome data	General risk of bias
Pittler et al. 1999	U	H	H	H	L	U	L	Bad
Schiller et al. 2001	U	H	H	H	L	U	L	Bad
Ho et al. 2001	U	H	H	H	L	U	H	Bad
Zahorska‐Markiewicz et al. 2002	U	L	H	H	L	H	L	Bad
Woodgate et al. 2003	L	H	L	H	L	U	L	Fair
Bokura and Kobayashi et al. 2003	L	L	H	H	L	U	L	Fair
Metso et al. 2003	U	L	H	H	L	U	L	Fair
Mhurchu et al. 2004	U	L	H	H	L	U	H	Bad
Lehtimäki et al. 2005	U	L	H	H	L	U	L	Fair
Kaats et al. 2006	U	L	H	L	L	U	H	Good
Liao et al. 2007	U	H	H	H	H	H	L	Bad
Hernández‐González et al. 2010	L	H	H	H	L	U	L	Bad
Willers et al. 2012	L	L	H	L	L	U	L	Good
Jung et al. 2014	U	L	H	H	L	H	L	Bad
Trivedi et al. 2015	U	L	H	L	H	H	L	Bad
Pokhis et al. 2015	L	L	H	L	L	U	L	Good
Santas et al. 2017	L	L	H	H	L	U	L	Fair
Cornelli et al. 2017	U	L	H	L	L	U	L	Good
Lütjohann et al. 2018	U	L	H	H	L	U	H	Bad

*Note:* General risk of bias: good < 2 high risk.

General risk of bias: fair = 2 high risk.

General risk of bias: bad > 2 high risk.

### 3.4 Qualitative Data Assessment

We judged the qualitative data based on the Cochrane risk‐of‐bias assessment tool. Five studies had a fair risk of bias (Bokura and Kobayashi 2003; Lehtimäki et al. 2005; Metso et al. 2003; Santas, Lázaro, and Cuñé 2017; Woodgate and Conquer 2003). Four studies had a poor risk of bias (Cornelli et al. 2017; Kaats, Michalek, and Preuss 2006; Pokhis et al. 2015; Willers, Plötz, and Hahn 2012). Eleven studies had a high risk of bias (Hernández‐González et al. 2010; Ho et al. 2001; Jung et al. 2014; Liao et al. 2007; Lütjohann et al. 2018; Mhurchu et al. 2004; Pittler et al. 1999; Schiller et al. 2001; Trivedi et al. 2015; Zahorska‐Markiewicz et al. 2002).

### 3.5 Effect of Chitosan Supplementation on Body Weight

**FIGURE 2:**
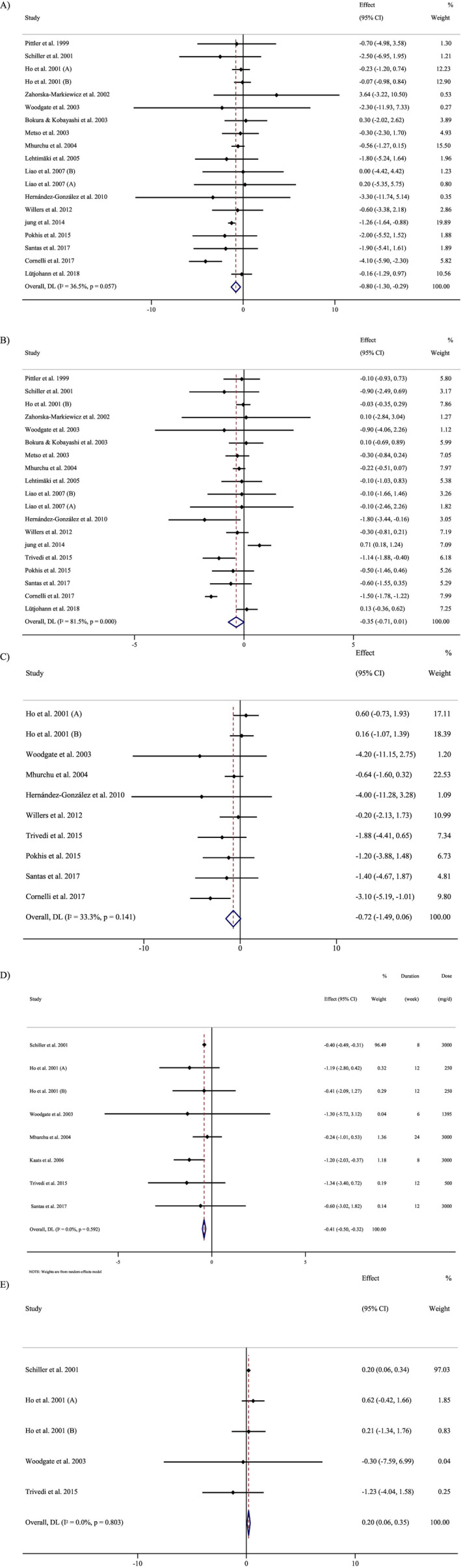
Forest plot detailing WMD and 95% CIs for the effect of chitosan consumption on (A) weight (kg), (B) BMI (kg/m^2^), (C) WC (cm), (D) BFP (%), and (E) FFM (kg).

Combining 19 effect sizes, including 1601 subjects (790 cases and 811 controls), manifested that chitosan supplementation had a significant inverse effect on body weight [WMD = −0.79 kg; 95% CI, −1.30 to −0.29; *p* = 0.002; (*I* ^2^ = 36.5%, *p* = 0.057)] (Figure 2A). According to subgroup analyses, chitosan supplementation had a reducing effect on body weight in all subgroups except at low supplementation doses (Table 3).

**TABLE 3:** Subgroup analyses of chitosan on anthropometric and body composition indices in adults.

	Number of effect size	WMD (95% CI)	*p*	Heterogeneity		
				*p* heterogeneity	*I* ^2^	*p* between subgroups
*Subgroup analyses of chitosan on weight (kg)*						
Overall effect	19	‐0.79 (−1.30, −0.29)	**0.002**	0.057	36.5%	
Trial duration (week)						
< 12	7	−1.21 (−1.58, −0.84)	**< 0.001**	0.851	0.0%	0.282
≥ 12	12	−0.78 (−1.47, −0.09)	**0.026**	0.039	46.4%	
Intervention dose (mg/day)						
< 1500	9	−0.16 (−0.77, 0.44)	0.592	0.997	0.0%	0.037
≥ 1500	10	−1.17 (−1.91, −0.44)	**0.002**	0.020	54.2%	
Baseline BMI (kg/m^2^)						
Overweight (25–29.9)	9	−0.77 (−1.25, −0.29)	**0.002**	0.304	15.5%	0.427
Obese (> 30)	9	−1.29 (−2.49, −0.09)	**0.034**	0.024	54.6%	
*Subgroup analyses of chitosan on BMI (kg/m* ^*2*^ *)*						
Overall effect	19	−0.35 (−0.71, 0.00)	0.054	< 0.001	81.5%	
Trial duration (week)						
< 12	7	0.22 (−0.17, 0.61)	0.265	0.374	7.1%	0.013
≥ 12	12	−0.50 (−0.91, −0.08)	**0.019**	< 0.001	85.2%	
Intervention dose (mg/day)						
< 1500	9	−0.31 (−0.65, 0.02)	0.070	0.161	32.1%	0.964
≥ 1500	10	−0.32 (−0.88, 0.22)	0.247	< 0.001	88.8%	
Baseline BMI (kg/m^2^)						
Overweight (25–29.9)	8	−0.00 (−0.29, 0.29)	0.989	0.209	27.6%	0.032
Obese (> 30)	10	−0.67 (−1.21, −0.13)	**0.014**	< 0.001	84.8%	
*Subgroup analyses of chitosan on WC (cm)*						
Overall effect	10	−0.71 (−1.49, 0.05)	0.069	0.141	33.3%	
Intervention dose (mg/day)						
< 1500	6	−0.09 (−0.94, 0.75)	0.825	0.351	10.1%	0.089
≥ 1500	4	−1.37 (−2.57, −0.17)	**0.025**	0.218	32.4%	
Baseline BMI (kg/m^2^)						
Overweight (25–29.9)	3	0.23 (−0.63, 1.10)	0.591	0.532	0.0%	0.021
Obese (> 30)	7	−1.22 (−2.09, −0.34)	**0.006**	0.323	14.0%	
*Subgroup analyses of chitosan on BFP (%)*						
Overall effect	8	−0.41 (−0.50, −0.32)	**< 0.001**	0.592	0.0%	
Trial duration (week)						
< 12	3	−0.66 (−1.28, −0.04)	**0.036**	0.160	45.4%	0.721
≥ 12	5	−0.50 (−1.10, 0.09)	0.096	0.773	0.0%	
Intervention dose (mg/day)						
< 1500	4	−0.96 (−1.94, 0.02)	0.056	0.887	0.0%	0.352
≥ 1500	4	−0.47 (−0.77, −0.17)	**0.002**	0.294	19.3%	
Baseline BMI (kg/m^2^)						
Overweight (25–29.9)	3	−0.77 (−1.82, 0.27)	0.146	0.796	0.0%	0.482
Obese (> 30)	4	−0.40 (−0.49, −0.30)	**< 0.001**	0.772	0.0%	
*Subgroup analyses of chitosan on FFM (kg)*						
Overall effect	5	0.20 (0.06, 0.34)	**0.005**	0.803	0.0%	
Trial duration (week)						
< 12	2	0.20 (0.05, 0.34)	**0.006**	0.893	0.0%	0.734
≥ 12	3	0.34 (−0.48, 1.17)	0.413	0.472	0.0%	
Baseline BMI (kg/m^2^)						
Overweight (25–29.9)	2	0.49 (−0.37, 1.35)	0.263	0.667	0.0%	0.506
Obese (> 30)	3	0.19 (0.05, 0.34)	**0.007**	0.604	0.0%	

*Note:* The bolded values are statistically significant (*p* < 0.05).

Abbreviations: BMI, body mass index; CI, confidence interval; FFM, fat‐free mass; FM, fat mass; WC, waist circumstance; WMD, weighted mean differences.

### 3.6 Effect of Chitosan Supplementation on BMI

In total, 19 effect sizes, including 1666 subjects (838 cases and 828 controls), were considered in this analysis. Pooled‐effect sizes showed no significant drop in BMI (kg/m^2^) by chitosan supplementation [WMD = −0.35 kg/m^2^, 95% CI: −0.71, 0.00; *p* = 0.054; (*I* ^2^ = 81.5%, *p* < 0.001)] (Figure 2B). Chitosan significantly reduced BMI (kg/m^2^) when obese participants (BMI > 30 kg/m^2^) were enrolled (Table 3).

### 3.7 Effect of Chitosan Supplementation on WC

In total, 10 effect sizes, including 796 subjects (414 cases and 382 controls), were considered in this analysis. Pooled‐effect sizes manifested no significant drop in WC by chitosan consumption (WMD = −0.71 cm, 95% CI: −1.49, 0.05; *p* = 0.069; *I* ^2^ = 33.3%, *p* = 0.141; Figure 2C). Based on the subgroup analyses, chitosan supplementation had a decreasing effect on WC when the intervention dose was ≥ 1500 mg/day and obese participants (BMI > 30 kg/m^2^) were enrolled (Table 3).

### 3.8 Effect of Chitosan Supplementation on BFP

Combining eight effect sizes from seven studies, including 641 subjects (337 cases and 304 controls), proposed that chitosan supplementation had a significant effect on BFP [WMD = −0.41%; 95% CI, −0.50 to −0.32; *p* < 0.001; (*I* ^2^ = 0.0%, *p* = 0.592)] (Figure 2D). Subgroup analyses demonstrated that chitosan supplementation had a significant decreasing effect on BFP when the trial duration was < 12 weeks, the intervention dose was ≥ 1500 mg/day, and obese participants (BMI > 30 kg/m^2^) were included (Table 3).

### 3.9 Effect of Chitosan Supplementation on FFM

Combining five effect sizes from four studies, including 247 subjects (141 cases and 106 controls), exhibited that chitosan supplementation had a significant effect on FFM [WMD = 0.20%; 95% CI, 0.06–0.34; *p* = 0.005; (*I* ^2^ = 0.0%, *p* = 0.803)] (Figure 2E). According to the subgroup analyses, chitosan supplementation had a significant increasing effect on FFM when trial duration was < 12 weeks and obese participants (BMI > 30 kg/m^2^) were included (Table 3).

### 3.10 Publication Bias

**FIGURE 3:**
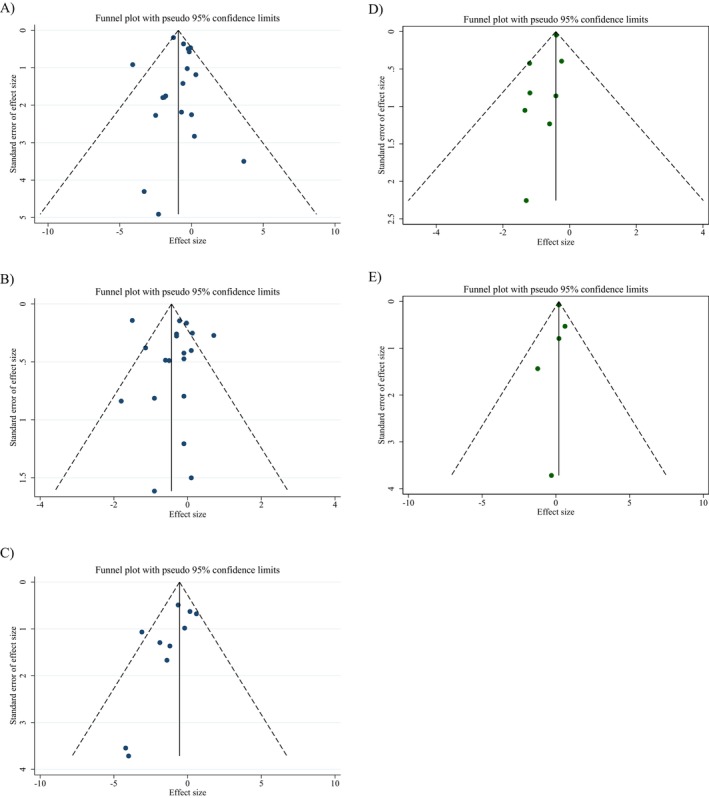
Funnel plots for the effect of chitosan consumption on (A) weight (kg), (B) BMI (kg/m^2^), (C) WC (cm), (D) BFP (%), and (E) FFM (kg).

Although small asymmetries were spotted during the visual inspection of funnel plots, no meaningful publication bias was detected for the weight (kg), BMI (kg/m^2^), WC, BFP, and FFM. The *p*‐values of Egger's test for body weight (*p*_Egger's test_ = 0.762, Figure 3A), BMI (*p*_Egger's test_ = 0.604, Figure 3B), WC (cm) (*p*_Egger's test_ = 0.068, Figure 3C), BFP (*p*_Egger's test_ = 0.106, Figure 3D), and FFM (*p*_Egger's test_ = 0.785, Figure 3E) did not exhibit significant publication bias.

### 3.11 Meta‐Regression Analysis

**FIGURE 4:**
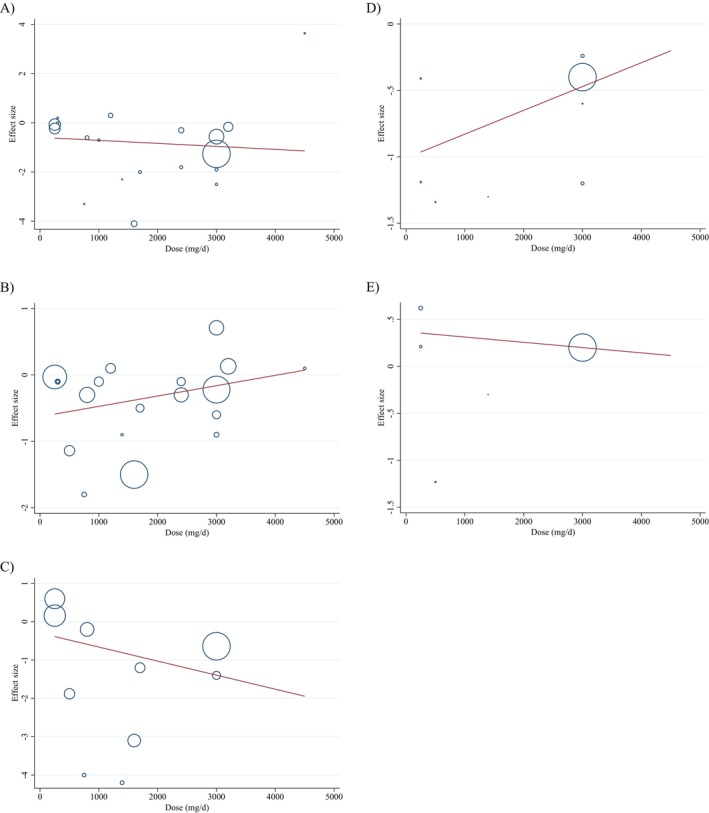
Linear dose–response relations between chitosan consumption and absolute mean differences. Dose–response relations between dose (mg/day) and absolute mean differences in (A) weight (kg), (B) BMI (kg/m^2^), (C) WC (cm), (D) BFP (%), and (E) FFM (kg).

**FIGURE 5:**
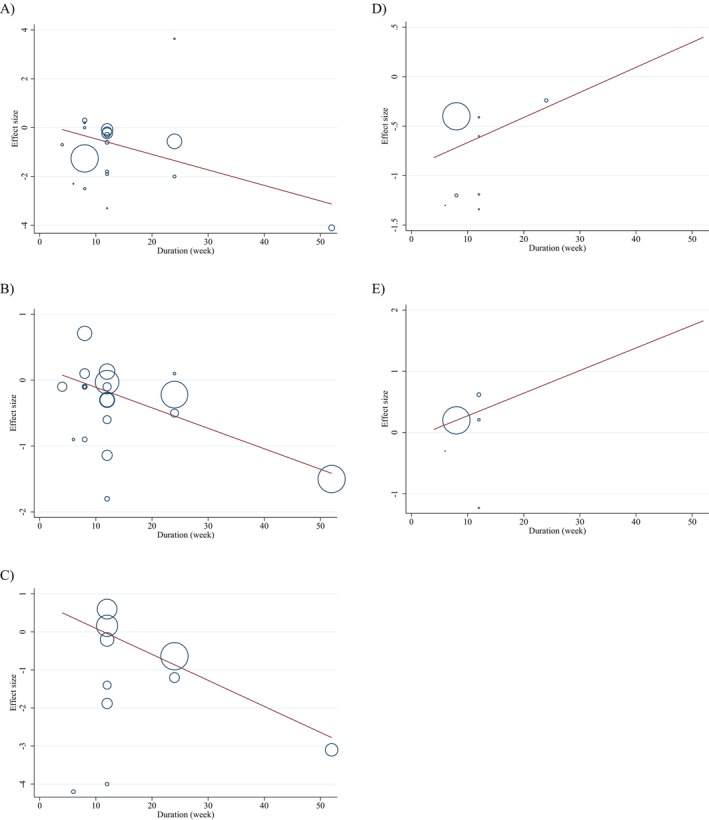
Linear dose–response relations between chitosan consumption and absolute mean differences. Dose–response relations between duration of intervention (week) and absolute mean differences in (A) weight (kg), (B) BMI (kg/m^2^), (C) WC (cm), (D) BFP (%), and (E) FFM (kg).

Meta‐regression analyses were directed to assess the potential impact of chitosan doses and intervention duration on body weight, BMI, WC, BFP, and FFM. There was no meaningful linear relationship between the dose of intervention (mg/day) and changes in body weight (coefficients = −0.00, *p* = 0.641), BMI (coefficients = 0.00, *p* = 0.300), WC (coefficients = −0.00, *p* = 0.379), BFP (coefficients = 0.00, *p* = 0.408), and FFM (coefficients = −0.00, *p* = 0.743) (Figure 4). There was a meaningful linear relationship between the duration of the intervention and changes in body weight (coefficients = −0.06, *p* = 0.021), BMI (coefficients = −0.03, *p* = 0.001), and WC (coefficients = −0.06, *p* = 0.033). However, there was no meaningful linear relationship between the duration of the intervention and changes in BFP (coefficients = 0.02, *p* = 0.528) and FFM (coefficients = 0.03, *p* = 0.752) (Figure 5).

### 3.12 Sensitivity Analysis

Based on the sensitivity analysis, the overall outcomes for weight, BFP, and FFM were not influenced by the exclusion of individual study effects. However, studies by Bokura and Kobayashi (2003) (WMD: −0.38, CI 95%: −0.75, −0.00), Jung et al. (2014) (WMD: −0.43, CI 95%: −0.78, −0.08), and Lütjohann et al. (2018) (WMD: −0.39, CI 95%: −0.77, −0.01) affected the overall results of BMI. Also, after removing study arms by Ho et al. (2001) (A) (WMD: −0.92, CI 95%: −1.69, −0.15) and Ho et al. (2001) (B) (WMD: −0.93, CI 95%: −1.82, −0.05), the overall outcomes of WC were altered.

### 3.13 Certainty Assessment

Table 4 presents the GRADE evidence profile and the level of certainty in the outcomes of chitosan supplementation related to weight, BMI, WC, BFP, and FFM. The evidence quality was deemed to be very high for weight, WC, BFP, and FFM. However, a serious limitation in inconsistency resulted in a moderate quality of evidence for BMI.

**TABLE 4:** GRADE profile of chitosan for anthropometric indices.

Outcomes	Risk of bias	Inconsistency	Indirectness	Imprecision	Publication bias	Quality of evidence
Weight	No serious limitation	No serious limitation	No serious limitation	No serious limitation	No serious limitation	⊕⊕⊕⊕ Very high
BMI	No serious limitation	Very serious limitation^a^	No serious limitation	No serious limitation	No serious limitation	⊕⊕◯◯ Moderate
WC	No serious limitation	No serious limitation	No serious limitation	No serious limitation	No serious limitation	⊕⊕⊕⊕ Very high
BFP	No serious limitation	No serious limitation	No serious limitation	No serious limitation	No serious limitation	⊕⊕⊕⊕ Very high
FFM	No serious limitation	No serious limitation	No serious limitation	No serious limitation	No serious limitation	⊕⊕⊕⊕ Very high

^a^ There is significant heterogeneity for BMI (*I* ^2^ = 81.1%).

## 4 Discussion

In the present meta‐analysis, we evaluated the chitosan effects on anthropometric and body composition indices as obesity indicators in adult population. Our findings showed that chitosan supplementation significantly reduced BFP and body weight. Also, FFM was significantly increased by chitosan consumption. Nevertheless, the results showed that chitosan supplementation had no significant effect on BMI and WC indices.

In the subgroup analysis, our results revealed that obesity status, trial duration, and dose of intervention had a significant effect on chitosan efficacy on anthropometric and body composition indices: when the dose of supplementation exceeded 2400 mg/day, the study duration was less than 12 weeks, and participants were either obese or overweight. In our investigation, we detected significant alterations in BMI and WC among obese individuals when the intervention lasted for 12 weeks or more. Conversely, when the intervention duration was under 12 weeks, notable effects on body composition parameters such as FFM and BFP were evident. In a systematic review and meta‐analysis conducted by Huang et al., it was shown that chitosan supplementation had significant effects on weight, BMI, and body fat compared to control groups, although its effects on WC were not significant (Huang et al. 2020). Subgroup analysis of this study showed that chitosan consumption had significant effects on body composition parameters when the dose of supplementation was more than 2400 mg/day, study duration was less than 12 weeks, and obese and overweight participants were enrolled. However, these results confirm our findings because in our study, when the duration of the intervention was less and more than 12 weeks and obese individuals were enrolled, significant effects on BMI and WC were observed, and also, when the duration of the intervention was less than 12 weeks, a significant effect on body composition parameters (FFM and BFP) was found. This little observed controversy might be explained by the different studies included in the Huang et al. meta‐analysis. Additionally, the number of RCTs (*n* = 6) included in our study was greater than that in the meta‐analysis by Huang et al., allowing for more accurate findings to be drawn from our results based on the dose‐response analysis. Chitosan may cause changes in anthropometric indices by affecting body composition, reducing the BFP, and also increasing the FFM percentage. Jull et al. conducted an extensive review that underscored chitosan's potential to significantly influence anthropometric indices, such as weight and BMI. This study also partially corroborates the findings of our meta‐analysis (Jull et al. 2008). However, our research diverges from the mentioned systematic review in terms of the comprehensiveness of our search strategy and the certainty and quality assessment of the included studies. Ernst et al. performed a meta‐analysis, demonstrating that the consumption of chitosan supplements over a 28‐day period resulted in an average reduction of 2.3 kg in body weight (Ernst and Pittler 1998). These outcomes align with our study, suggesting that the intake of chitosan for a duration of 12 weeks or longer can yield substantial effects on body weight. It is worth noting that Ernst et al. included a limited number of low‐quality studies (five articles), which could potentially influence their findings. Chitosan, derived from the acetylated chitin family, stands out as a non‐toxic and non‐allergenic compound increasingly incorporated into non‐prescription weight loss products (Ríos‐Hoyo and Gutiérrez‐Salmeán 2016). Numerous animal and clinical studies have delved into the potential mechanisms underlying chitosan's influence on energy homeostasis and body weight. Its impact on anthropometric indices and body composition stems from various mechanisms, include decreasing cholesterol absorption as an insoluble fiber, facilitated by its bile acid resin effect (van Bennekum et al. 2005), binding with fat molecules in the intestinal tract, thus impeding their absorption (Saper, Eisenberg, and Phillips 2004), modulating the secretion of adipokines, ultimately inhibiting lipogenesis (Rahman, Kumar, and Yun 2010), and lowering inflammatory factors like C‐reactive protein and elevating serum leptin levels, both contributing to effective weight loss (Pan and Myers Jr 2018; Walsh et al. 2013). Moreover, the effects of chitosan extend beyond its direct impact on body weight. Studies have indicated its potential in improving insulin sensitivity and glucose metabolism, which are crucial factors in mitigating the risk of developing type 2 diabetes mellitus (Yang et al. 2017). Additionally, chitosan supplementation has been associated with a reduction in triglyceride levels, thereby decreasing the risk of cardiovascular diseases (Wang and Chen 2014). Furthermore, emerging research suggests that chitosan may exert beneficial effects on gut microbiota composition and function. By promoting the growth of beneficial bacteria while inhibiting harmful pathogens, chitosan supplementation could contribute to overall gut health and potentially influence weight management (Li and Zhuang 2020; Xiao et al. 2019).

Chitosan emerges as a promising natural supplement for combating overweight and obesity and mitigating the risk of associated chronic diseases. Its multifaceted mechanisms of action, ranging from cholesterol reduction to modulation of adipokine secretion, underscore its potential as a valuable tool in promoting overall health and well‐being. Further research is warranted to elucidate its precise mechanisms and optimal dosing strategies for maximal efficacy in clinical settings.

Chitosan supplementation is believed to exert its effects on body weight and BMI through mechanisms such as reducing the rate of fat absorption (Hayashi and Ito 2002; Tai et al. 2000). Previous animal studies have suggested that combining chitosan supplementation with regular physical activity and a balanced diet may enhance its efficacy in promoting weight loss and improving anthropometric indicators (Sumiyoshi and Kimura 2006; Zalaqi et al. 2022). However, contrasting findings have been reported in a crossover study, wherein the administration of 1200 mg/day of chitosan over a 10‐month period did not yield a statistically significant impact on the body weight of individuals without underlying health conditions (Metso et al. 2003). This discrepancy underscores the complexity of chitosan's effects on body weight regulation and highlights the need for further research to elucidate the factors influencing its efficacy across different populations and contexts. Despite the divergent outcomes, it is essential to consider various factors such as dosage, duration, participant characteristics, and study design when interpreting the findings of studies investigating the effects of chitosan supplementation on body weight and anthropometric measures. Future studies should aim to address these factors comprehensively to provide a clearer understanding of the potential benefits and limitations of chitosan as a supplement for weight management.

The current study acknowledges several limitations, which may impact the interpretation of results. One notable limitation is the presence of an overall elevated risk of bias across the included studies. This potential bias could stem from various factors such as inadequate blinding, incomplete outcome data, and selective reporting, which may introduce uncertainty into the validity of the findings. Another limitation is the absence of an assessment of certain body composition and anthropometric parameters, including waist−hip ratio (WHR), waist−height ratio (WHtR), arm circumference (AC), and calf circumference (CC). These parameters are crucial indicators of overall health and adiposity distribution, and their inclusion could provide a more comprehensive understanding of the effects of chitosan supplementation on body composition. Furthermore, considering the substantial impact of dietary habits and physical activity levels on body composition and anthropometric indicators in both obese and non‐obese individuals, the exclusion of these factors from the analysis represents a limitation. Including diet and physical activity as subgroups in the analysis could enhance the generalizability and robustness of our findings by accounting for these influential variables. It is suggested that in the future studies that will be conducted on the effects of chitosan on body composition and anthropometric indices, these limitations should be removed so that the obtained data can be more easily generalized to the society and its validity can be increased.

Despite these limitations, the current study contributes valuable insights into the effects of chitosan supplementation on anthropometric indices. Future research efforts should aim to address these limitations by implementing rigorous study designs, incorporating comprehensive outcome assessments, and accounting for relevant confounding factors to strengthen the validity and generalizability of findings in this field. To the best of our knowledge, this study stands as one of the pioneering comprehensive systematic reviews and meta‐analyses aimed at assessing the effects of chitosan supplementation on anthropometric measurements and body composition in adult populations. By synthesizing data from a diverse range of studies, this research endeavors to provide a robust understanding of chitosan's potential impact on body weight and composition. A key strength of this study lies in the conducted subgroup analysis, which allows for a more nuanced examination of the effects of chitosan supplementation on the assessed parameters. This approach enables the identification of potential variations in outcomes based on factors such as dosage, duration of supplementation, and participant characteristics, thereby enhancing the depth and reliability of the findings. Furthermore, an additional notable strength of this study is the limited evidence of publication bias observed in the included literature. The comprehensive search strategy and meticulous inclusion criteria employed in this research minimize the risk of overlooking relevant studies, thus reducing the likelihood of bias in the synthesized evidence. By addressing these methodological considerations and leveraging robust statistical techniques, this study endeavors to provide a comprehensive and reliable assessment of the effects of chitosan supplementation on anthropometric measurements and body composition. The findings of this research hold significant implications for informing clinical practice and guiding future research efforts in this field.

## 5 Conclusions

The findings of the current systematic review and meta‐analysis suggest that chitosan supplementation has a significant impact on reducing body weight and BFP, as well as increasing FFM in adult individuals. Moreover, subgroup analysis revealed significant effects of the prolonged duration of chitosan supplementation (more than 12 weeks) in reducing BMI and WC, as well as the effective dose of chitosan supplementation (more than 1500 mg/day) in reducing WC. Combining chitosan consumption with a healthy lifestyle may serve as an adjunctive therapy and complementary treatment strategy for obese or overweight individuals. However, further well‐designed studies with larger sample sizes are necessary to confirm these conclusions.

## Author Contributions

**Mona Kholdebarin:** writing – original draft preparation. **Naseh Pahlavani:** writing – original draft preparation. **Mahlagha Nikbaf‐Shandiz:** software, validation. **Halle Mosallaei:** writing – original draft preparation. **Niloufar Rasaei:** data curation. **Omid Asbaghi:** conceptualization, methodology. **Yasaman Aali:** data curation. **Farideh Shiraseb** and **Ali Zamanian:** writing – original draft preparation. **Zeinab Khalse** and **Ali Zamanian:** writing – reviewing and editing. **Ali Zamanian:** revised the manuscript.

## Ethics Statement

The authors have nothing to report.

## Conflicts of Interest

The authors declare no conflicts of interest.

## Data Availability

Data will be available upon request.
